# CXCL12/CXCR4 pathway is activated by oncogenic JAK2 in a PI3K-dependent manner

**DOI:** 10.18632/oncotarget.10789

**Published:** 2016-07-22

**Authors:** Hadjer Abdelouahab, Yanyan Zhang, Monika Wittner, Shinya Oishi, Nobutaka Fujii, Rodolphe Besancenot, Isabelle Plo, Vincent Ribrag, Eric Solary, William Vainchenker, Giovanni Barosi, Fawzia Louache

**Affiliations:** ^1^ INSERM, UMR 1170, Gustave Roussy, Villejuif, France; ^2^ University Paris Diderot, Paris, France; ^3^ University Paris-Sud 11, Villejuif, France; ^4^ Gustave Roussy, Villejuif, France; ^5^ Center for the Study of Myelofibrosis, Biotechnology Research Area, IRCCS Policlinico S. Matteo Foundation, Pavia, Italy; ^6^ Kyoto University, Graduate School of Pharmaceutical Sciences, Kyoto, Japan; ^7^ Equipe labellisée Ligue Nationale contre le Cancer, UMR 1170, Institut Gustave Roussy, Villejuif, France; ^8^ Grex, Laboratoire d’Excellence, Paris, France

**Keywords:** JAK2 inhibitors, CXCR4, hematopoiesis

## Abstract

JAK2 activation is the driver mechanism in *BCR-ABL-*negative myeloproliferative neoplasms (MPN). These diseases are characterized by an abnormal retention of hematopoietic stem cells within the bone marrow microenvironment and their increased trafficking to extramedullary sites. The CXCL12/CXCR4 axis plays a central role in hematopoietic stem cell/ progenitor trafficking and retention in hematopoietic sites. The present study explores the crosstalk between JAK2 and CXCL12/CXCR4 signaling pathways in MPN. We show that JAK2, activated by either MPL-W515L expression or cytokine stimulation, cooperates with CXCL12/CXCR4 signaling to increase the chemotactic response of human cell lines and primary CD34^+^ cells through an increased phosphatidylinositol-3-kinase (PI3K) signaling. Accordingly, primary myelofibrosis (MF) patient cells demonstrate an increased CXCL12-induced chemotaxis when compared to controls. JAK2 inhibition by knock down or chemical inhibitors decreases this effect in MPL-W515L expressing cell lines and reduces the CXCL12/CXCR4 signaling in some patient primary cells. Taken together, these data indicate that CXCL12/CXCR4 pathway is overactivated in MF patients by oncogenic JAK2 that maintains high PI3K signaling over the threshold required for CXCR4 activation. These results suggest that inhibition of this crosstalk may contribute to the therapeutic effects of JAK2 inhibitors.

## INTRODUCTION

*BCR-ABL1* negative myeloproliferative neoplasms (MPN), i.e. Polycthemia Vera (PV), Essential Thrombocythemia (ET) and Primary Mylofibrosis (PMF) are clonal disorders occurring in hematopoietic stem cells (HSC). The most frequent mutation is *JAK2*V617F, which is associated with the three *BCR-ABL* negative MPNs, whereas mutations of *CALR* in exon 9 and *MPL*W515 (usually *MPL*W515L or *MPL*W515K) are only associated with ET and PMF [1, 2].

All MPN driver mutations (*JAK2*V617F, *MPL*W515L/K, *CALR* mutations) activate the cytokine/receptor JAK2 pathway and its downstream signaling such as the Signal transducer and activator of transcription (STAT1, 3 and 5), phosphatidylinositol 3-kinase (PI3K)/AKT/mTOR and the MAPK/ extracellular signal-regulated kinase (ERK) pathways. These mutations are responsible for myeloproliferation and can induce myelofibrosis (MF) in mouse models [3–8]. However, other mutations may be present that are targeting epigenetic regulators and the spliceosome that favor clonal dominance and/or modify disease phenotype to induce MF and/or progression to leukemia [9–11].

While normal HSC and progenitor cells (HSPC) proliferate and differentiate in the bone marrow microenvironment, patients with MPN are characterized by extramedullary hematopoiesis (EMH) that takes place in various sites with a marked prominence in the spleen. [12, 13] Recent data have documented the existence of MF HSC residing in the spleen with increased transplantation capacity in comparison to blood or marrow HSC [14]. Cues promoting this MF HSC circulation and homing to extramedullary territories have not been completely characterized. Interestingly, a recent study has shown that the MF splenic environment is characterized by an increased level of intact and functional CXCL12 that can contribute to the localization of MF CD34^+^ cells to the spleen [15].

CXCR4, the receptor for CXCL12, is a master regulator of cell trafficking in normal and pathological settings [16–18]. CXCR4 promotes HSC retention within the BM microenvironment [19, 20], and CXCR4 inhibitors induce neutrophil and HSC mobilization [21, 22]. These cells are mobilized from BM, but also from EMH sites [23]. Similarly, CXCR4 antagonists specifically target malignant leukemic precursors, present both in BM and EM tissues [24, 25]. These data are consistent with CXCL12 expression in BM and extramedullary organs [26, 27], and indicate that CXCL12/CXCR4 axis is involved in the dynamic interplay between HSC and multiple different tissue compartments.

Several alterations of the CXCL12/CXCR4 axis have been identified in MF, including the abnormal processing of CXCL12 in a pathological environment [28] and a decreased expression of CXCR4 through hypermethylation of the gene promoter [29–31]. Nevertheless, MF CD34^+^ cells demonstrate higher *in vitro* migration compared to normal control peripheral blood (PB) CD34^+^ cells [32]. Thus, even in a context of low CXCR4 expression, a gain of function of CXCR4 characterizes MF CD34^+^ cells, which may favor their maintenance within the bone marrow microenvironment and extramedullar sites. It is presently unknown whether there is a crosstalk between JAK2 oncogenic activation and CXCL12/CXCR4 signaling, which may be involved in cell trafficking and EMH.

Here, we show that JAK2 activation by both oncogenic events and exogenous cytokines synergize with CXCL12/CXCR4 pathway to induce chemotaxis and signaling via PI3K activation. Altogether, these results suggest that JAK2 inhibitors can reduce cell trafficking by decreasing the CXCL12/CXCR4 activity. This may be part of their therapeutic activity, which mainly targets EMH. Moreover, these data provide a rationale to explore the therapeutic activity of combined therapy between JAK2 inhibitors and CXCR4 antagonists or PI3K inhibitors.

## RESULTS

### MPLW515L expression increases the chemotactic response to CXCL12

**Figure 1:**
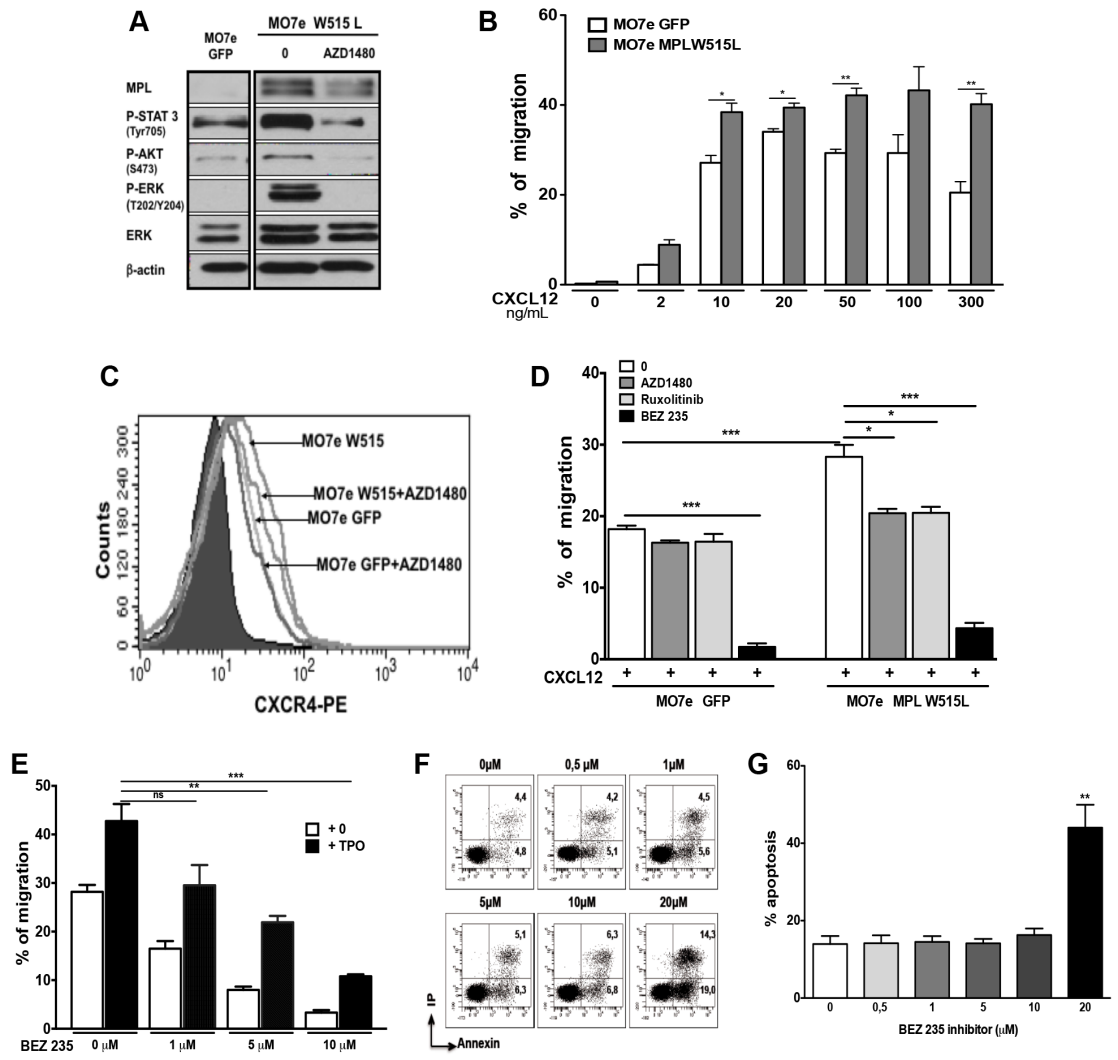
Constitutive activation of JAK2 and downstream pathways by MPLW515L enhance the chemotactic response of MO7e cells to CXCL12 MO7e cells were transduced with retroviral vectors expressing GFP alone or GFP with MPLW515L gain-of-function mutation. **A**. Investigation by western-blot of MPL expression and downstream signaling pathway (STAT3, AKT and ERK) activation in MO7e- MPLW515L and mock-transduced cells (MO7e-GFP) in the cells without starvation. Treatment with AZD1480 (2 μM) inhibits the constitutive phosphorylation of STAT3, AKT and ERK. **B**. Effect of MPLW515L expression on CXCL12-induced migration in transwell assay. Migration assay data obtained for control MO7e cells were pooled with those obtained when TPO effects were tested (Figure 2A). **C**. MPL W515L expression in MO7e cells and AZD1480 (2 μM) inhibitor do not modify CXCR4 membrane expression. **D**. AZD1480 (2 μM), Ruxolitinib (2 μM) or BEZ235 (10 μM) strongly inhibited chemotaxis of mutant cells to CXCL12 (100 ng/ml). **E**. Effects of the dual PI3K/mTOR inhibitor BEZ235 on MO7e cell migration in response to CXCL12. MO7e cells were treated with various concentrations (1, 5 or 10 μM) of BEZ235 for 2 hours. TPO (10 ng/mL) was then added or not and the migration in response to CXCL12 (100 ng/mL) was measured in chemotactic assay. The data shown represent the mean ± SEM of 3 independent experiments. **F**. Treatment with 10 μM of BEZ235 inhibitor does not induce apoptosis in human MO7e cells. After treatment with the indicated concentrations of BEZ235 for 6 hours, MO7e cells were stained with Annexin-V and propidium iodide. The percentages of Annexin-V positive cells were determined by flow cytometry. **G**. Effects of BEZ235 inhibitor on the viability of MO7e cells. Columns represent the mean of 3 independent experiments. Data are represented as mean ± SEM.

To investigate a possible crosstalk between JAK2 and CXCR4 signaling, we expressed the gain-of-function *MPL*W515L mutation in MO7e cells, a cytokine-dependent cell line. MO7e-MPLW515L cells were selected for cytokine-independent growth and displayed constitutive STAT3, PI3K and ERK phosphorylation that were reverted by the JAK2/JAK1 inhibitor AZD1480 (Figure 1A). As compared to mock-transduced cells, MO7e-MPLW515L cells showed increased migratory response to CXCL12 (Figure 1B), which was not correlated with an increase in CXCR4 membrane expression (Figure 1C). This enhanced migratory response was reverted to control level by pretreatment of cells for 2 hours with, AZD1480 or Ruxolitinib, two selective JAK1 and JAK2 inhibitors (Figure 1D). In contrast, JAK inhibition had only a weak effect on mock-transduced MO7e cells (Figure 1D). Of note, CXCR4 membrane expression was not modified by MPLW515L expression or JAK1/2 inhibition (Figure 1C).

The importance of the PI3K/AKT pathway in cell migration induced by CXCL12 was investigated by analysing the effects of different doses of BEZ 235 (a dual PI3K/AKT inhibitor). As shown in Figure 1E, MO7e cells treatment with BEZ235 for 2 hours induced a dose-dependent inhibition of CXCL12-induced migration with a maximal inhibition at 20 μM. However, as 20 μM resulted in significant apoptosis (Figure 1F and 1G), we used 10 μM as a working concentration in subsequent studies. At this concentration, cell chemotaxis was completely inhibited in both MO7e-GFP and MO7e-MPLW515L cells (Figure 1D). Thus, the chemotaxis to CXCL12 of MO7e control and MO7e-MPLW515L cells was dependent on the PI3K pathway triggered through CXCR4 signaling. This chemotaxis was increased by oncogenic JAK2 activation without changes in CXCR4 expression.

### Diverse cytokines enhance the chemotactic response to CXCL12 in MO7e cells

**Figure 2:**
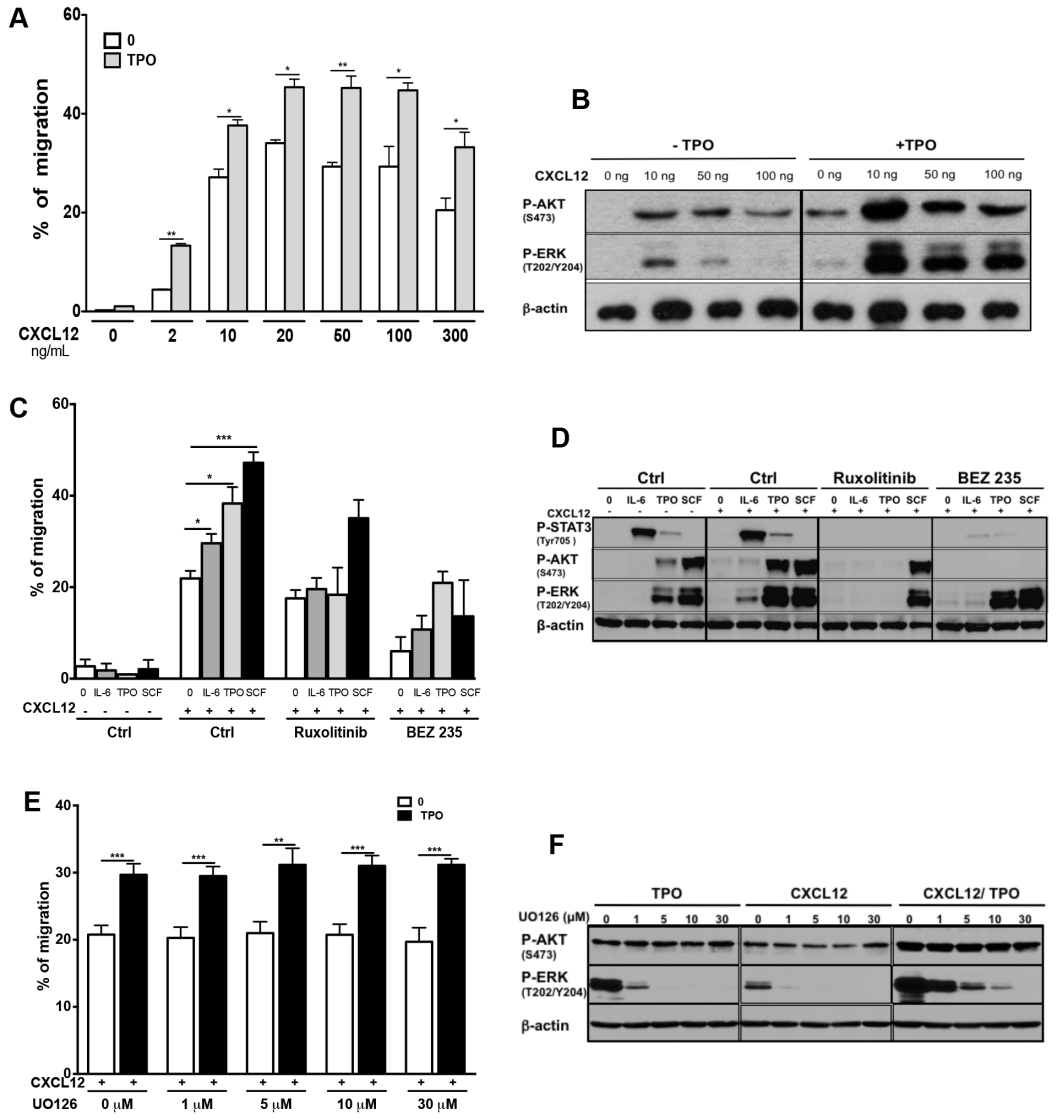
Cytokine-mediated activation of signaling pathways enhances the chemotactic response of MO7e cells to CXCL12 **A**. Effect of TPO on CXCL12-mediated chemotaxis in MO7e-GFP control cells. After starvation, cells were treated or not with 10 ng/mL TPO and chemotaxis was performed in the presence of different concentrations of CXCL12. Migration assay data obtained for control MO7e-GFP cells were pooled with those obtained when the effect of MPLW515L was tested (Figure 1B). **B**. Investigation by Western-blot of AKT and ERK activation by CXCL12 and TPO. Cells were treated or not with TPO in the presence of different concentrations of CXCL12 for 10 minutes before lysate preparation. **C**. Effects of Ruxolitinib and BEZ235 on CXCL12-induced migration of MO7e cells upon stimulation with IL-6 (10 ng/mL), TPO (10 ng/mL) or SCF (25 ng/mL). Chemotaxis was assayed in the absence or presence of CXCL12 (100 ng/mL). **D**. Phosphorylation of STAT3, AKT and ERK in MO7e pretreated or not with Ruxolitinib (2 μM) or BEZ235 (10 μM) before stimulation with CXCL12 (100 ng/mL) alone, cytokines alone (IL-6, TPO, SCF) or combinations of both for 10 minutes. Cells were serum and cytokine starved for 4 hours prior cytokine addition. **E**. Effects of UO126 inhibitor treatment on MO7e cell migration in response to CXCL12.MO7e cells were starved and treated with various concentrations (1, 5, 10 or 30 μM) of UO126 for 2 hours. TPO (10 ng/mL) was then added or not and the migration in response to CXCL12 (100 ng/mL) was measured in chemotactic assay. The data shown represent the mean ± SEM of 3 independent experiments. **F**. Investigation by Western-blot of AKT and ERK activation by CXCL12 alone, TPO alone or combination of both in MO7e cells, after pre-incubation with various concentrations of UO126.

To determine whether cytokine activation of JAK2 pathway has similar effects as MPLW515L, we studied if TPO could increase the migratory response of MO7e cells to CXCL12. Migration towards CXCL12 was significantly augmented by TPO at all CXCL12 concentrations used (Figure 2A). Western Blot experiments showed that CXCL12-induced phosphorylation of AKT and ERK was further enhanced by the addition of TPO (Figure 2B). Ruxolitinib abrogated the enhancing effect of TPO, whereas BEZ235 nearly completely inhibited MO7e migration in response to CXCL12 (Figure 2C). To determine whether this effect was restricted to TPO, we stimulated MO7e cells with interleukin-6 (IL-6) which signals through JAK1/JAK2, and with Stem Cell Factor (SCF) that signals through c-KIT. Both cytokines markedly increased CXCL12-induced MO7e migration (Figure 2C). Pretreatment of MO7e cells with Ruxolitinib (2 μM) for 2 hours completely inhibited the effects of IL-6, while demonstrating little inhibition of SCF-induced cell migration. Pretreatment of MO7e cells with BEZ235 (10 μM) decreased the synergistic effect of IL6, SCF and TPO on CXCL12-induced cell migration. The combination of each cytokine with CXCL12 triggered an increased activation of AKT and ERK and to a lesser extent of STAT3 (Figure 2D). In these experiments, AKT and ERK activation by CXCL12 alone was not observed because it occurred before 10 minutes [35, 36]. Pretreatment with Ruxolitinib abrogated AKT, ERK and STAT3 activation by TPO and IL-6 but not by SCF. In contrast, the pretreatment of cells with BEZ235 abrogated STAT3 and AKT, but not ERK activation (Figure 2D). The ERK1/2 MAPK signalling integrates extracellular cues that induce proliferation and differentiation. As this pathway is also involved in the migration of some cell types [37, 38], we also tested the effects of the MEK1/2 inhibitor UO126. We observed that UO126 (from 1 to 30 μM) had no effect on CXCL12-induced migration of MO7e cells (Figure 2E), whereas it abrogated ERK phosphorylation induced by TPO, CXCL12 or both (Figure 2F). These results suggest that PI3K signaling is the major pathway involved in CXCL12-induced chemotaxis of MO7e cells which could be further activated by the cytokine receptor/JAK2 or tyrosine kinase receptor pathways.

**Figure 3:**
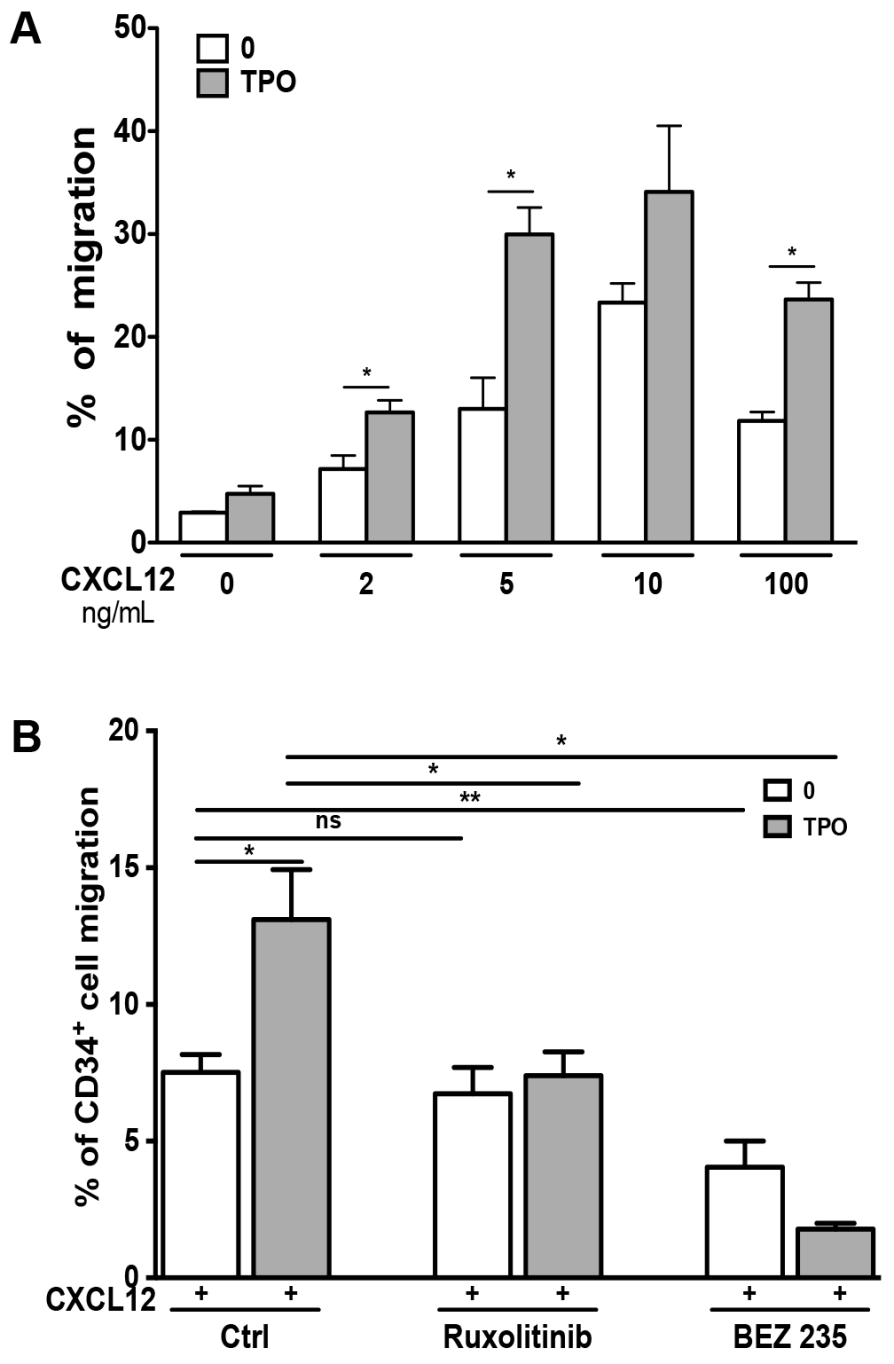
Cytokine-mediated activation of signaling pathways enhances the chemotactic response of primary CD34+ cells to CXCL12 **A**. Effect of TPO on CXCL12/CXCR4 signaling in CD34^+^ cells. TPO (10 ng/mL) was added or not to the upper chamber and the migration in response to CXCL12 (100 ng/mL) was measured in chemotactic assay. **B**. Starved CD34^+^ cells were pre-incubated for 2 hours with Ruxolitinib (2 μM) or BEZ235 (10 μM) before stimulation or not with TPO (10 ng/mL). Chemotaxis was then performed in the presence of CXCL12 (100 ng/mL). Data are represented as mean ± SEM.

This synergy between CXCL12 and TPO in inducing MO7e cell migration was also observed in primary CD34^+^ cells (Figure 3A). Again, Ruxolitinib specifically inhibited the effect of TPO, whereas BEZ235 nearly completely abrogated the chemotactic effect of the TPO and CXCL12 combination (Figure 3B).

**Figure 4:**
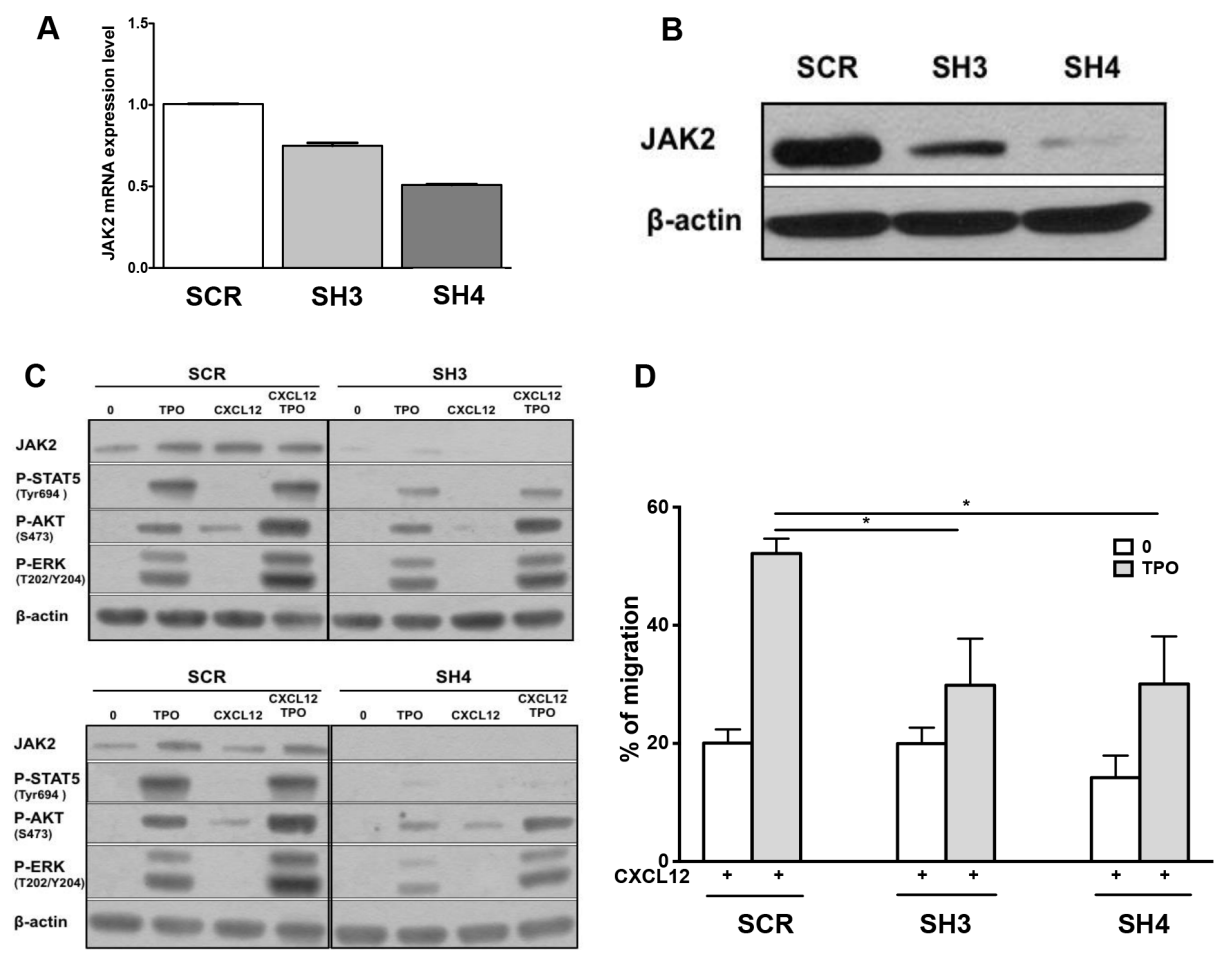
Effects of shRNAs against JAK2 WT on the chemotactic response of MO7e cells to CXCL12 MO7e cells were transduced with two different shRNAs against JAK2 WT (SH3 and SH4) and a control scramble shRNA (SCR). **A**. Effects of the two shJAK2 (SH3 and SH4) on *JAK2* transcripts were evaluated by qRT-PCR. **B**. Investigation by Western-blot of JAK2 protein expression upon transduction with the two shJAK2. **C**. Effects of the two shJAK2 (SH3 and SH4) on the phosphorylation of STAT5, AKT and ERK by TPO alone, CXCL12 alone or by combination of both. **D**. Chemotactic response to CXCL12 (100 ng/mL) of MO7e cells expressing control scramble SCR, SH3 or SH4 shRNA, in the absence or presence of TPO (10 ng/mL). Data are represented as mean ± SEM.

To exclude an off-target effect of JAK inhibitors, we chose to knock down JAK2 using a RNA interference strategy. Among 4 sequences tested, two sequences SH3 and SH4 decreased JAK2 transcripts by 26% and 50% respectively, as shown by QRT-PCR (Figure 4A). Moreover, SH3 and SH4 exhibited an efficient effect at the protein level with 77% and 95% of inhibition, respectively (Figure 4B). Both shRNAs and more particularly SH4 inhibited the phosphorylation of STAT5, AKT and ERK by TPO and by the combination of CXCL12 and TPO (Figure 4C). In addition, both shJAK2 inhibited the stimulatory effects of TPO on chemotaxis of MO7e cells to CXCL12 (Figure 4D), demonstrating a crosstalk between JAK2 activation and CXCR4 signaling.

### Phosphoinositide-3-kinase isoforms differentially regulate the synergistic effect of JAK2 activation on cell migration

**Figure 5:**
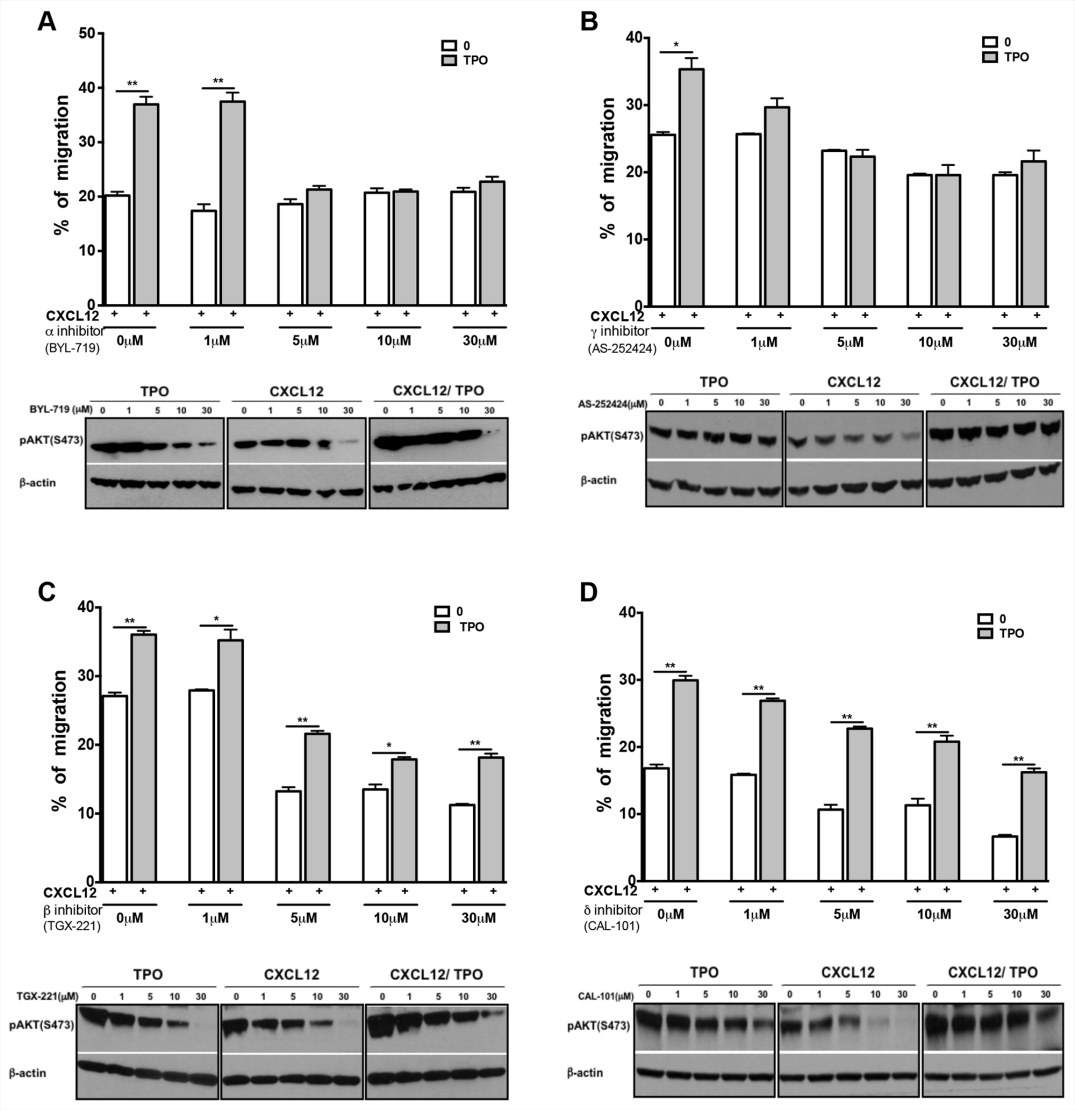
PI3K α and PI3K γ inhibitors abolish the cooperative effect induced by TPO and CXCL12 MO7e cells were treated for 2 hours with various concentrations (1, 5, 10 or 30 μM) of PI3K inhibitors. **A**. PI3K α inhibitor (BYL-719), **B**. PI3K γ inhibitor (AS-252424), **C**. PI3K β inhibitor (TGX-221), **D**. PI3K δ inhibitor (CAL-101). TPO (10 ng/mL) was then added or not and chemotaxis was performed in the presence of CXCL12 (100 ng/mL). Data are represented as mean ± SEM.

PI3Ks isoforms have been classified into 3 groups according to their structure and substrate specificity. Class IA isoforms are comprised of one of p85 regulatory subunits and a catalytic subunit (p110) that has four isoforms (p110 α, β, δ and γ). The activity of these isoforms couples to receptor tyrosine kinases (class Ia) or to G protein–coupled receptors (class Ib) [35]. To determine which PI3K isoform is involved in the collaborative effect of CXCL12 and JAK2 activation on cell migration, we assessed the ability of diverse PI3K subunit inhibitors to prevent chemotaxis of MO7e cells exposed to CXCL12. MO7e cells were treated with various concentrations of PI3K α, γ, β or δ inhibitors for 2 hours before migration assays. All PI3K inhibitors except PI3K α (BYL-719) had a small but significant inhibitory effect on CXCL12-induced chemotaxis of unstimulated MO7e cells (Figure 5A, 5B, 5C, 5D). This was correlated with a reduction in AKT phosphorylation that was particularly evident at 10 μM. PI3K α (BYL-719) and γ (AS-252424) but not β (TGX-221) and δ (CAL-101) inhibitors abrogated the collaboration between CXCL12 and TPO (Figure 5). In line with this, the collaborative effect between TPO and CXCL12 was also abrogated at the level of AKT phosphorylation (Figure 5). Altogether, these results suggest that the PI3K β and δ subunits mediate CXCL12-induced migration, whereas PI3K α and γ mediate the amplification of the CXCL12/CXCR4 axis effects by JAK2 activating cytokines.

### MF CD34^+^ cells demonstrate increased chemotaxis to CXCL12 that is reverted by genetic ablation of JAK2

**Figure 6:**
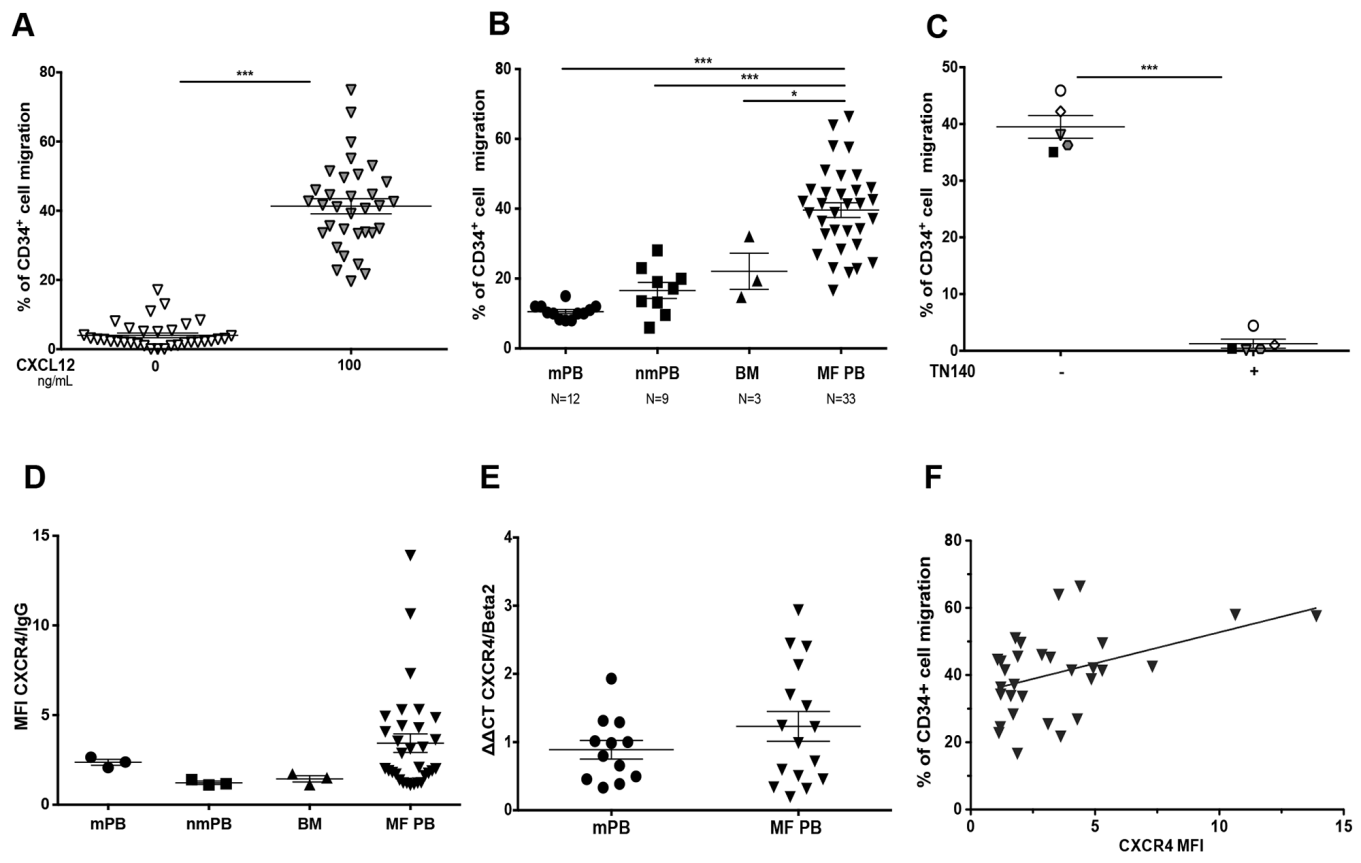
Chemotaxis of MF CD34+ cells in response to CXCL12 **A**. PB CD34^+^ cells from 33 MF patients were assayed for chemotaxis in the absence and presence of CXCL12 (100 ng/ml). **B**. CD34^+^ cells from mobilized peripheral blood (mPB), non-mobilized PB (nmPB) and bone marrow (BM) samples were assayed for CXCL12-induced chemotaxis compared to MF CD34^+^ cells. **C**. CD34^+^ cells from MF patients were pre-incubated or not with TN140 (5 μM) for 30 min before chemotaxis assays. **D**. CXCR4 membrane expression and the MFI was determined on gated CD45^+^/CD34^+^ cells from mobilized PB (mPB), non-mobilized PB (nmPB), BM and MF PB samples. **E**. CXCR4 mRNA expression (relative to *β2 microglobulin*) was quantified by qRT-PCR in CD34^+^ cells from mobilized and MF patients. **F**. Correlation between chemotaxis and the overall CXCR4 expression was determined on gated CD45^+^/CD34^+^ MF cells. Data are represented as mean ± SEM.

To test whether our results are relevant to MF patients, the *in vitro* migratory responses to CXCL12 of PB CD34^+^ cells were analyzed in 33 MF patients (20 PMF, 6 post-ET/MF and 7 post-PV/MF). An increased chemotactic response to CXCL12 was noticed in MF CD34^+^ cell samples (Figure 6A) which was in average greater than that observed with CD34^+^ cells from different controls (mobilized PB, non-mobilized PB and BM) (Figure 6B). However, a heterogeneous migratory response to CXCL12 was noticed, as some patient samples migrated in a similar manner as the controls. Pretreatment of MF CD34^+^ cells with the CXCR4 inhibitor TN140 inhibited CXCL12-induced migration (Figure 6C) demonstrating that this migratory response is CXCR4 specific. CXCR4 expression was heterogeneous among MF CD34^+^ cells (Figure 6D, 6E) and was slightly, but significantly correlated with the chemotactic response (r=0.4994, P= 0.0068) (Figure 6F). Thus, MF CD34^+^ cells exhibit a strong chemotactic response to CXCL12 even in case of low or intermediate CXCR4 membrane expression, suggesting that the CXCL12/CXCR4 signaling pathway is over activated in MF patients.

**Figure 7:**
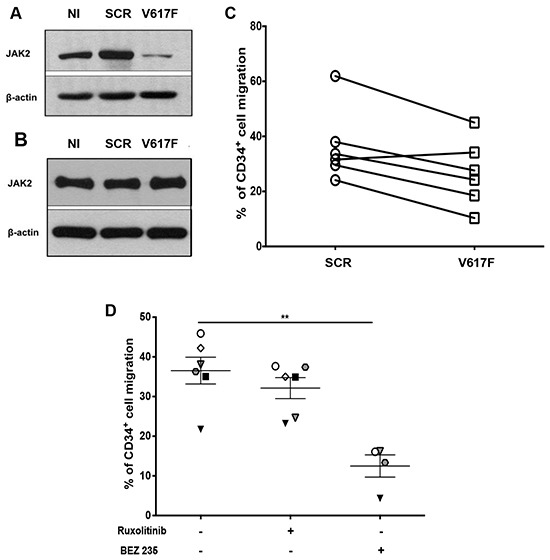
Effect of JAK2 and PI3K inhibition on chemotaxis of MF CD34+ cells in response to CXCL12 **A**. Jak2 protein level as revealed by Western-blot in HEL cells that express JAK2V617F. HEL cells were either non-infected (NI) or infected with control scramble (SCR) or V617F shRNA. A 95% reduction of the JAK2 protein was obtained with the V617F shRNA. **B**. JAK2 protein level was analyzed by Western-blot in MO7e cells that express the wild type form of JAK2. MO7e cells were either non-transduced (NI) or transduced with control scramble (SCR) or V617F shRNA. Results show a selective and specific inhibition of JAK2V617F protein expression present in HEL with shV617F, but not of the wild type form of JAK2 present in MO7e. **C**. Chemotactic responses to CXCL12 (100 ng/mL) of MF CD34^+^ cells expressing control scramble (SCR) or V617F shRNA. **D**. Effects of Ruxolitinib and BEZ235 inhibitors on MF CD34^+^ cell migration in response to CXCL12. Data are represented as mean ± SEM.

To determine the role of oncogenic JAK2, we specifically knocked down JAK2V617F in MF CD34^+^ cells using a specific shRNA against JAK2V617F. The specificity of this shRNA was assessed by showing that it knocked down JAK2 in HEL cells, which only express JAK2V617F (Figure 7A), but not in MO7e cells that only express JAK2WT (Figure 7B). In 5 out of 6 JAK2V617F MF patients, the shJAK2V617F decreased chemotaxis to CXCL12 compared to shSCR-GFP-expressing cells (Figure 7C). These results indicate that JAK2V617F signaling is involved in the CXCL12-mediated migration of MF CD34^+^ cells in most cases.

To assess whether JAK inhibitors interfere with CXCL12/CXCR4 axis in MF, we performed migration experiments with CD34^+^ cells from 6 MF patients in the absence or presence of Ruxolitinib (Figure 7D). Ruxolitinib inhibited CXCL12-induced migration in 3 out of 6 samples. This result with those previously observed with the shJAK2V617F underscores the heterogeneity of MF patients. Pretreatment of MF CD34^+^ cells with 10 μM of the pan-PI3K inhibitor BEZ235 resulted in a significant reduction of chemotaxis in response to CXCL12 (Figure 7D), further illustrating the role of PI3K in cell migration.

## DISCUSSION

Constitutive oncogenic activation of JAK2 signaling leads to an aberrant proliferation, survival and differentiation of cells [39]. However, few studies have examined the impact of JAK2 activation on the migration and trafficking of hematopoietic progenitors.

Here, we explored whether oncogenic JAK2 could increase CXCR4 signaling. Using MO7e cells transduced with MPLW515L, we have shown that JAK2 activation strongly collaborates with CXCR4 signaling. This crosstalk increased CXCL12-dependent migration and further activation of STAT, PI3K and ERK pathways. A similar result was observed for primary human CD34^+^ or MO7e cells when stimulated by cytokines that require JAKs for signaling. JAK2 inhibition or knock down abolished this cooperation. Interestingly, JAK2 inhibition had no effect on CXCL12-induced chemotaxis of unstimulated CD34^+^ or MO7e cells.

Recent studies indicate that JAK inhibition induces significant reduction in splenomegaly and symptomatic improvement in MPN patients, without induction of complete remission and eradication of the malignant clone [40]. Moreover, a growing body of evidence suggests that CXCL12 production is involved in EMH. Therefore, our results showing that JAK2 inhibition reduces CXCL12-induced chemotaxis in JAK2 stimulated cells, may explain why the major effect of JAK2 inhibitors is mainly on spleen reduction and not on allele burden.

Previous studies have shown that CXCL12 could activate JAK2, suggesting that JAK2 is involved in CXCL12/CXCR4-mediated migration [41]. However, these studies were performed using low specificity JAK2 kinase inhibitors and were not confirmed when more specific JAK2 inhibitors or a genetic silencing approach were used. Indeed, further studies showed that JAK2 is involved in the integrin activation induced by CXCL12/CXCR4 signaling [42, 43]. Altogether, these studies demonstrate that JAK2 activation is not directly involved in cell migration, but increases CXCL12/CXCR4-induced chemotaxis.

The mechanism involved in the cooperative effect between CXCR4 and JAK2 signaling appears to be related to the level of PI3K activation. Two lines of evidence support this hypothesis: 1) phosphorylation of AKT and ERK was highly increased by the simultaneous addition of CXCL12 and a cytokine, and 2) a PI3K inhibitor completely abolished chemotaxis induced by CXCL12 alone or in association with cytokines, whereas a MEK1/2 inhibitor had no effect. Furthermore, we showed that this increased migration was related to the activation of different PI3K isoforms: class IA by cytokine receptors and class IB by CXCR4. Thus, normal migration in response to CXCL12 requires PI3K activity. This observation is in line with previous data showing that PI3K activity is an indispensable signaling event that is required for cells to undergo chemotaxis [44]. In addition, pathologic migration in response to CXCL12 requires a high level of PI3K activity. Indeed, JAK2 inhibitors will decrease the activation of PI3K under the threshold required for CXCL12/CXCR4-induced chemotaxis.

Previous studies have shown that CD34^+^ cells of MF patients exhibit abnormal trafficking during disease development leading to EMH predominantly in the red pulp of the spleen. It has been suggested initially that the major mediator for the EMH is the fibrotic bone marrow environment and more recently the role of inflammatory cytokines has been underscored [45]. Our results indicate that cell-intrinsic mechanisms may also be involved, as MF CD34^+^ cells displayed increased migration in response to CXCL12. CXCR4 promotes HSC retention within the BM microenvironment [19, 20] and its genetic invalidation or inhibition results in increased HSC mobilization [21, 22]. In normal hematopoiesis, this cell mobilization derives primarily from BM. Interestingly, during malignancies, CXCR4 antagonists target malignant leukemic precursors present in BM and EM tissues [24, 46]. In line with this, increased concentrations of CXCL12 were found in the spleen of MF patients, the site where MF HSC reside [15]. More recently, it has been shown that two niche factors SCF and CXCL12 are responsible for EMH [47]. Our results point out the possibility that EMH in the spleen from MPN patients is in part due to an increased activation of the CXCL12/CXCR4 pathway. In this study, we also show that one of the roles of SCF consists to increase the chemotaxis induced by CXCL12/CXCR4. This may contribute to the threshold levels of PI3K activation, which are required for CXCR4 effects. Thus, we can hypothesize that the oncogenic activation of JAK2 favors EMH by diminishing the requirement for SCF in the niche. Further experiments will be required to test this hypothesis.

On the other hand, we observed that the JAK2 inhibitor Ruxolitinib inhibited chemotaxis at least for some patients. This could be related to the heterogeneity of MF, as pointed out by the large spectrum of additional mutations found in this disease. Alternatively, Ruxolitinib may have limited effects on PI3K activation by JAK2V617F or MPLW515L [48, 49]. In this case, it is possible that the new type 2 JAK inhibitors might be more effective by inhibiting more drastically oncogenic JAK2 activation.

In a previous study, we have demonstrated that simultaneous inhibition of JAK2 and PI3K signaling pathways led to delayed splenomegaly in mice inoculated with Ba/F3-MPL/JAK2V617F cells. This suggests that targeting both JAK2 and the PI3K pathway with PI3K/AKT/mTOR inhibitors will be of interest in the treatment of patients [48]. However, such treatment may increase toxicity on hematopoiesis and induce profound cytopenia. Thus, it might be interesting to use JAK2 inhibitors together with class IB PI3K inhibitors that will preserve hematopoiesis or to directly target CXCR4 with CXCR4 antagonists. These approaches may also affect leukemic initiating cells by profoundly altering their interaction with the extramedullary niche. MPN mouse modelling of the disease will be important to understand the precise role of this increased CXCL12/CXCR4 activity in the development of EMH and whether spleen reduction by JAK2 inhibitor is mainly mediated by induction of mobilization.

## MATERIALS AND METHODS

### Patient and control samples

PB samples from patients who fulfilled the WHO diagnostic criteria for PMF or post PV/ET MF were collected in accordance with the Declaration of Helsinki. They were obtained from patients that had given their informed written consent following protocols approved by local Research Ethics Committees from Gustave Roussy Institute (Villejuif, France).

### Cell lines

MO7e cells were transduced with MSCV-*hMPLW515L*-IRES-GFP and selected by their growth in the absence of GM-CSF (10 ng/mL) in alpha-MEM medium (Invitrogen) supplemented with 10% fetal bovine serum. For chemotaxis assay and western blotting (WB), cells were starved and treated for 2 hours at 37°C with 2 μM Ruxolitinib, 10 μM BEZ235 or with various concentrations (1, 5, 10 or 30 μM) of PI3K α (BYL-719), γ (AS-252424), β (TGX-221), δ (CAL-101) inhibitors or MEK1/2 inhibitor (UO126) (Euromedex, Souffelweyersheim, France).

### CXCR4 expression analysis

Mononuclear cells were triple-stained with an APC-conjugated anti-CD34 monoclonal antibody (mAb) (HPCA2), a fluorescein FITC-conjugated anti-human CD45 mAb and a PE-conjugated anti-CXCR4 mAb (12G5). Appropriate Ig isotype were used as controls. All antibodies were from BD. Cells were analyzed by a FACSort cytometry (BD Biosciences). Quantitative analysis of fluorescence intensity was performed on gated CD34^+^ cells and the mean fluorescence intensity (MFI) was calculated using Cell Quest software package. Mean fluorescence intensity ratios (MFIRs) were calculated by dividing the MFI of CXCR4 by the MFI of the respective nonspecific isotype control.

### Quantitative real-time PCR

RNA extraction was done using a Qiagen RNA isolation kit (Qiagen). All PCR reactions were carried out using Taqman PCR Master Mix (Applied Biosystems). Each cDNA sample was analyzed in triplicate in the ABI Prism 7900 Sequence Detection System. The sequences of primers used are: CXCR4-Forward (5′-CGTGCCCTCCTGCTGACTATT-3′), CXCR4-Reverse (5′-GCCAACCATGATGTGCTGAA-3′), CXCR4-Probe (5′-TTCATCTTTGCCAACGTCAGTGAGGCA-3′), JAK2-Forward, (5′-AAGCTTTCTCACAAGCATTTGGTTT-3′), JAK2-Reverse (5′-AGAAAGGCATTAGAAAGCCTGTAGTT-3′), JAK2-Probe (5′-TCTCCACAGACACATAC-3′), β2M-Forward (5′-GACTTTGTACAGCCCAAGATA-3′), β2M-Reverse (5′-GCGGCATCTTCAAACCTCC-3′), β2M-Probe (5′-TGATGCTGCTTACATGCTTCGATCCCACTT-3′).

### Chemotaxis assay

Migration assays were performed in serum-free medium (serum-free Dulbecco's medium supplemented with 1.5% bovine serum albumin (BSA; Cohn's fraction V; Sigma), sonicated lipids, and iron-saturated human transferrin), using 5-μm pore filter chambers (Transwell, ThinCert 24 well Translucent RoTrac, Greiner Bio-one) as previously described [33]. Briefly, cells were resuspended in serum-free medium to a final concentration of 1 × 10^6^ cells/mL, and 100 μL of the cell suspension were placed into the upper chamber, whereas 600 μL of serum-free medium without or with CXCL12 (Peprotech, France) at different concentrations were placed in the lower compartment. In some experiments, cells were stimulated with 10 ng/mL TPO (Kirin Brewery, Tokyo, Japan) before chemotaxis assay. For chemotaxis blocking studies, inhibitors were added to the cell suspension 60 mn before migration assays. The transwell plates were incubated for 3 hours at 37°C in 5% CO_2_ and 95% air. Cells recovered from lower chambers were counted by flow cytometry. All assays were done in triplicate. Data are presented as percentage of migration calculated by the following ratio: the number of cells migrating to the lower chamber/ the number of cells used for migration assay. Outside the effect-dose studies, the concentration of CXCL12 used in migration experiments was 100 ng/ml for the sake of consistency with studies using primary CD34^+^ cells from normal and MF patients.

### Lentiviral and retroviral vector construction

Two short hairpin RNAs (shRNA) directed against the wild type form of JAK2 (SH3 and SH4), one shRNA directed against JAK2V617F and one shRNA unrelated to JAK2 (SCR: scramble control sequence) were inserted into a pBlue Script containing the human *H1* promoter. The H1-shJAK2WT/V617F or H1-SCR cassettes were inserted into a lentiviral vector (pRRLsin-PGK-eGFP; Genethon, Evry, France) [34]. The oligonucleotides used in this study were the following: SH3: 5′-GCTTTGTCTTTCGTGTCATTA-3′, SH4: 5′-GAGTATGTGTCTGTGGAGA-3′, shJAK2V617F:5′-GAGTATGTTTCTGTGGAGA-3′, shSCR containing a scramble sequence:5′- CGGCAGCTAGCGACGCCAT-3.

### Production of retroviruses and lentiviruses

Vesicular stomatitis virus glycoprotein pseudotyped viral particles were produced in 293T cells. Cells were infected with lentiviral supernatants for 2 hours and GFP positive cells were sorted 48 hours later by flow cytometry (FACSDiva; BD Biosciences, Mountain View, CA).

### Annexin-V assay

Annexin-V positive staining was determined by FACS analysis according to the manufacturer's recommendations (BD Pharmingen, Franklin Lakes, NJ, USA).

### Western blotting

Signaling studies were performed using rabbit antibodies directed against MPL, JAK2 (Tyr1007/ 1008) and the phosphorylated forms of STAT3 (Tyr705), STAT5 (Tyr694), extracellular signal-regulated kinase 1/2 (ERK1/2) (Thr202/Tyr204) and AKT (Ser473) (Cell Signaling Technology). Anti β-actin antibody from Sigma (Saint Quentin Fallavier, France) was used to control equal loading.

### Statistical analysis

Results given as mean ± S.E.M were analyzed with the two-tailed unpaired Student's *t*-test. Differences were considered significant when P-value was *, p<0.05; **, p<0.01 and ***, p<0.001.
